# Impacts of heart rate variability on post-traumatic stress disorder risks after physical injuries: amplification with childhood abuse histories

**DOI:** 10.3389/fpsyt.2024.1474650

**Published:** 2024-12-19

**Authors:** Ji Hyeon Jeon, Ju-Wan Kim, Hee-Ju Kang, Hyunseok Jang, Jung-Chul Kim, Ju-Yeon Lee, Sung-Wan Kim, Il-Seon Shin, Jae-Min Kim

**Affiliations:** ^1^ Department of Psychiatry, Chonnam National University Medical School, Gwangju, Republic of Korea; ^2^ Division of Trauma, Department of Surgery, Chonnam National University Medical School and Hospital, Gwangju, Republic of Korea

**Keywords:** post-traumatic stress disorder, child abuse, heart rate variability, risk factors, longitudinal study

## Abstract

**Introduction:**

This study examined the moderating effects of childhood abuse histories on the associations between low frequency (LF) and high frequency (HF) components of heart rate variability (HRV) and the development of post-traumatic stress disorder (PTSD).

**Methods:**

Participants with physical injuries were recruited from a trauma center and followed for two years. Baseline assessments included LF, HF, and childhood abuse histories, assessed using the Nemesis Childhood Trauma Interview. Socio-demographic and clinical covariates were obtained. PTSD diagnoses were made at 3, 6, 12, and 24 months post-injury using the Clinician-Administered PTSD Scale for DSM-5. Logistic regression analyses assessed the associations.

**Results:**

Among 538 participants, 58 (10.8%) developed PTSD during the study period. A significant interaction was found: lower LF/HF were significantly associated with PTSD in patients with childhood abuse histories, but not in those without.

**Conclusion:**

Childhood abuse history significantly moderates the relationship between LF-HF HRV components and PTSD development, suggesting that childhood adversities amplify the risk. These findings support the importance of screening for childhood abuse histories and monitoring HRV in physically injured patients as part of the assessment process.

## Introduction

Post-traumatic stress disorder (PTSD) is a severe psychiatric condition marked by a spectrum of psychological and physiological symptoms that significantly impair daily functioning and quality of life. The autonomic nervous system (ANS), which regulates physiological responses to stress, is integral to the development and persistence of PTSD symptoms. Heart Rate Variability (HRV) is a well-established physiological marker that evaluates ANS function, indicating the balance between sympathetic and parasympathetic nervous system activities (1). Lower HRV often signifies reduced cardiovascular adaptability, heightening vulnerability to PTSD (2).

HRV is typically examined through its spectral components, particularly the low frequency (LF) and high frequency (HF) bands. The LF band (0.04-0.15 Hz) reflects both sympathetic and parasympathetic activity, whereas the HF band (0.15-0.40 Hz) predominantly indicates parasympathetic activity and is frequently influenced by respiratory patterns (3). These spectral components offer crucial insights into the autonomic regulation of heart rate and have been extensively researched concerning PTSD.

Extensive research, including several meta-analyses, has demonstrated significant links between low HRV (both LF and HF components) and increased PTSD risk (1, 4–7). These results suggest that individuals with diminished HRV may have a reduced capacity to manage stress, thereby heightening their susceptibility to PTSD. However, some studies have reported inconsistent or non-significant correlations of LF and HF components with PTSD (8–11). These inconsistencies underscore the complexity of this relationship and the necessity for further exploration to identify factors influencing these associations.

Given these mixed results, it is crucial to examine potential moderating factors that might explain the variability in the HRV-PTSD relationship. One such factor is a history of childhood abuse, which has been extensively linked to both ANS dysregulation and the development of PTSD. Studies have shown that individuals with a history of childhood abuse often exhibit reduced HRV, reflecting impaired autonomic regulation and increased stress vulnerability (12, 13). Additionally, childhood abuse has been associated with a heightened risk of developing PTSD, as it can lead to lasting changes in brain structure and function, affecting emotional regulation and stress responses (14, 15). It can thus be hypothesized that the impact of HRV on PTSD risk may be moderated by early-life adversities. Despite this plausible link, no studies to date have explored the moderating effect of childhood abuse histories on the HRV-PTSD relationship.

This study aims to investigate the moderating role of childhood abuse histories on the association between HRV (LF and HF components) and the development of PTSD over a two-year period. Specifically, we seek to determine how these relationships can inform the clinical application of HRV monitoring as a predictive tool in identifying individuals at heightened risk of PTSD. By integrating HRV measurements with assessments of childhood abuse histories, our objective is to enhance the predictive accuracy of PTSD risk assessments, potentially guiding targeted interventions and preventive strategies in clinical settings.

## Methods

### Study overview and participants

This analysis is part of the Biomarker-based Diagnostic Algorithm for Post-Traumatic Syndrome (BioPTS) study, aimed at improving PTSD diagnostic and predictive models. Detailed protocols are provided in a prior publication (16). The study prospectively enrolled patients admitted to the Trauma Center at Chonnam National University Hospital (CNUH), South Korea, between June 2015 and January 2021. Inclusion criteria were: i) age 18 or older at the time of injury; ii) hospitalization for more than 24 hours following a moderate to severe physical injury (Injury Severity Score, ISS ≥9) (17); and iii) proficiency in Korean to understand the study protocol. Exclusion criteria included: i) moderate or severe brain injury (Glasgow Coma Scale, GCS <10) (18); ii) injuries resulting from suicide attempts; iii) severe physical conditions preventing comprehensive psychiatric evaluation; iv) history of psychiatric disorders (excluding depression and anxiety); v) significant cognitive impairments due to organic or neurocognitive disorders; and vi) pre-existing convulsive disorders or anticonvulsant use. Baseline assessments, including HRV measures and childhood abuse histories, were conducted within one month of hospitalization. The average duration from injury to baseline assessment was 8.8 (± 5.3) days. Follow-up evaluations were conducted via telephone at 3, 6, 12, and 24 months post-injury using the Clinician-Administered PTSD Scale for DSM-5 (CAPS-5) (19). The CNUH Institutional Review Board approved the study (CNUH 2015-148), and informed consent was obtained from all participants.

### HRV data collection and analysis

HRV data, specifically LF and HF components, were obtained using the SA-6000 HRV analyzer (Medicore Co., Seoul, Korea). Participants rested for 5 minutes before testing, removed any metal accessories, kept their eyes open, and lay comfortably. To minimize movement or posture-related biases, participants remained still, breathing naturally without speaking. Electrode sensors were placed on both wrists and the left ankle, and a 3-minute recording was taken. After ensuring the proper setup for HRV recording, it was essential to control for factors that could influence the reliability of our results. To mitigate potential confounding effects such as medication use, body position, and time of day, we standardized the conditions under which HRV measurements were taken as much as possible, given the clinical context of severe physical injury. Medication was not restricted, as it was crucial for the immediate well-being of the participants. All HRV assessments were conducted in the morning to minimize variability caused by diurnal factors. Additionally, since our participants were receiving emergency treatment, all recordings were made while patients were in a supine position to ensure consistency across assessments. A trained experimenter supervised to ensure adherence to the protocol. LF and HF (ms²) parameters were derived using the Medicore HRV Analysis System. Due to the lack of established reference values, LF and HF data were dichotomized using median values.

### Childhood abuse

Childhood abuse was assessed using the Nemesis Childhood Trauma Interview (20). In this semi-structured interview, participants were asked if they had experienced emotional or psychological, physical, and sexual abuse before the age of 16. Emotional abuse was evaluated with questions such as, “Were you emotionally or psychologically abused, meaning being yelled at, falsely punished, subordinated to your siblings or being blackmailed?”; physical abuse by asking, “Were you physically abused, meaning being hit, kicked, beaten up, or experiencing other types of physical abuse?”; and sexual abuse by inquiring, “Were you sexually abused, meaning being touched or having to touch someone in a sexual way or being pressured into sexual contact against your will?”. Since these forms of abuse often co-occur (21), a broad definition of “childhood abuse” (experiencing at least one type of abuse) was used for the analysis.

### Other baseline characteristics

To comprehensively assess factors potentially influencing PTSD development and HRV outcomes, various baseline characteristics were documented.

#### Socio-demographic characteristics

Data included age, sex, education duration, marital status (married or not), living status (living alone or not), and employment status (employed or not).

#### Pre-trauma characteristics

Histories of psychiatric disorders (depression, panic disorder, agoraphobia, social phobia, generalized anxiety disorder) were recorded. Lifetime traumatic events were assessed using the Life Events Checklist (22). Physical disorders were screened through a comprehensive questionnaire. Smoking status (current smoker or not) and alcohol use (AUDIT score) (23) were documented, along with Body Mass Index (BMI).

#### Trauma related characteristics

The type of traumatic injury was categorized using the Life Events Checklist (22) to distinguish between unintentional and intentional injuries. Injury severity was assessed with ISS and GCS scores.

#### Peri-trauma characteristics

PTSD symptom severity was measured using CAPS-5, while anxiety and depression were assessed with the Hamilton Anxiety Rating Scale (HAMA) (24) and the Hamilton Depression Rating Scale (HAMD) (25), respectively. Baseline vital signs, including blood pressure (BP) and heart rate, were recorded.

### Follow-up diagnoses of PTSD

The CAPS-5, a reliable and valid tool for PTSD assessment, was used for follow-up evaluations. Participants met DSM-5 criteria across symptom clusters, including symptom duration and functional significance. PTSD diagnosis was confirmed at 3, 6, 12, and 24 months post-trauma.

### Statistical analysis

Participants who completed at least one follow-up were included in the analysis. Baseline characteristics were compared using t-tests or χ² tests. LF and HF data were transformed logarithmically for analysis, a common practice in HRV research to normalize the distribution (26). Logistic regression analyzed the individual associations of LF, HF, and childhood abuse histories with PTSD development, adjusting for significant covariates. The modifying effects of childhood abuse histories on the LF-HF and PTSD relationship were assessed using multinomial logistic regression with interaction terms. To ensure the validity of the logistic regression models, we addressed key statistical assumptions. The linearity of the logit for each continuous variable was assessed using the Box-Tidwell procedure. Additionally, to examine potential multicollinearity among the variables, we calculated Variance Inflation Factor (VIF) values for each covariate. All tests were two-sided with a significance level of P < 0.05, conducted using SPSS version 27.0 and STATA version 18.

## Results

### Recruitment and baseline data

The recruitment process and PTSD prevalence are depicted in **Supplementary Figure S1**. Out of 1,142 patients initially assessed at baseline, 580 (50.8%) underwent HRV evaluation. **Supplementary Table S1** compares the baseline characteristics between those who completed the HRV evaluation and those who did not. Higher ISS scores were significantly associated with non-completion of HRV assessment, while other variables showed no significant differences. Among the patients who completed the HRV assessment, 42 (7.4%) did not continue beyond the 3-month evaluation, resulting in a final cohort of 538 patients (92.6%) for analysis. There were no significant differences in baseline characteristics between those who completed the study and those who did not (all P-values > 0.05). Within this cohort, 58 patients (10.8%) were diagnosed with PTSD over the two-year period. Comparisons of baseline characteristics between patients with and without PTSD are detailed in **Supplementary Table S2**. Significant factors associated with PTSD diagnosis included female sex, higher education levels, previous psychiatric disorders, prior traumatic events, and elevated anxiety and depressive symptoms. Lower HF (≤ 38 ms²) was significantly related to older age, higher anxiety and depressive symptoms, and increased heart rate (**Supplementary Table S3**). Additionally, childhood abuse histories were significantly associated with younger age, fewer physical disorders, current smoking status, and higher AUDIT scores. However, no significant associations were found between childhood abuse histories and LF and HF HRV components (**Table 1**). Based on these analyses and collinearity considerations, nine covariates were selected for further analysis: age, sex, previous psychiatric disorders, prior traumatic events, number of physical disorders, current smoking status, AUDIT scores, HAMD scores, and heart rate.

**Table 1:** Baseline characteristics by childhood abuse (CA) histories in 538 patients with physical injuries.

	Absent CA (N=501)	Present CA (N=37)	Statistical coefficients	P-value^a^
Socio-demographic characteristics				
Age, mean (SD) years	57.7 (16.7)	47.5 (17.5)	t=+3.570	**<0.001**
Sex, N (%) female	162 (32.3)	7 (18.7)	χ^2^=2.879	0.090
Education, mean (SD) years	10.7 (4.3)	10.9 (3.2)	t=-0.478	0.629
Marital status, N (%) unmarried	163 (32.5)	15 (40.5)	χ^2^=0.997	0.318
Living alone, N (%)	77 (15.4)	2 (5.4)	χ^2^=2.730	0.098
Unemployed status, N (%)	80 (16.0)	8 (21.6)	χ^2^=0.805	0.370
Pre-trauma characteristics				
Previous psychiatric disorders, N (%)	36 (7.2)	5 (13.5)	χ^2^=1.960	0.162
Previous traumatic events, N (%)	26 (5.2)	3 (8.1)	χ^2^=0.575	0.448
Physical disorders, mean (SD) numbers	2.1 (2.1)	1.3 (1.6)	t=+2.782	**0.008**
Current smoker, N (%)	123 (24.6)	17 (45.9)	χ^2^=8.193	**0.004**
AUDIT, mean (SD) scores	9.9 (9.8)	14.7 (10.9)	t=-2.854	**0.004**
Body mass index, mean (SD)	23.7 (3.5)	23.1 (3.1)	t=+0.991	0.322
Trauma related characteristics				
Injury type, N (%) intentional	48 (9.6)	3 (8.1)	χ^2^=0.087	0.768
Injury Severity Score, mean (SD) scores	14.1 (5.3)	13.5 (5.0)	t=+0.576	0.565
Glasgow Coma Scale, mean (SD) scores	14.9 (0.6)	14.8 (0.8)	t=+0.381	0.703
Got surgery for the injury, N (%)	246 (49.1)	22 (59.5)	χ^2^=1.479	0.224
Peri-trauma assessment scales and measurements, mean (SD)				
HAMA, scores	4.7 (4.7)	5.2 (4.9)	t=-0.662	0.508
HAMD, scores	6.2 (5.4)	7.4 (5.8)	t=-1.248	0.212
Systolic blood pressure, mmHg	119.2 (14.0)	118.4 (15.8)	t=+0.309	0.757
Diastolic blood pressure, mmHg	72.0 (9.2)	71.0 (9.0)	t=+0.622	0.534
Heart rate per minute	78.7 (11.1)	78.9 (10.5)	t=-0.142	0.887
Heart rate variability measures, log transformed mean (SD) ms2				
Low frequency band	4.0 (1.3)	4.4 (1.4)	t=-1.665	0.108
High frequency band	3.5 (1.5)	3.8 (1.4)	t=-1.249	0.212

a t-tests or χ^2^ tests, as appropriate between patients with and without 24-months follow-up evaluation.

AUDIT, Alcohol Use Disorders Identification Test; HAMA, Hamilton Anxiety Rating Scale; HAMD, Hamilton Depression Rating Scale. Bold values indicate statistical significance at P < 0.05.

### Individual associations

**Figure 1:**
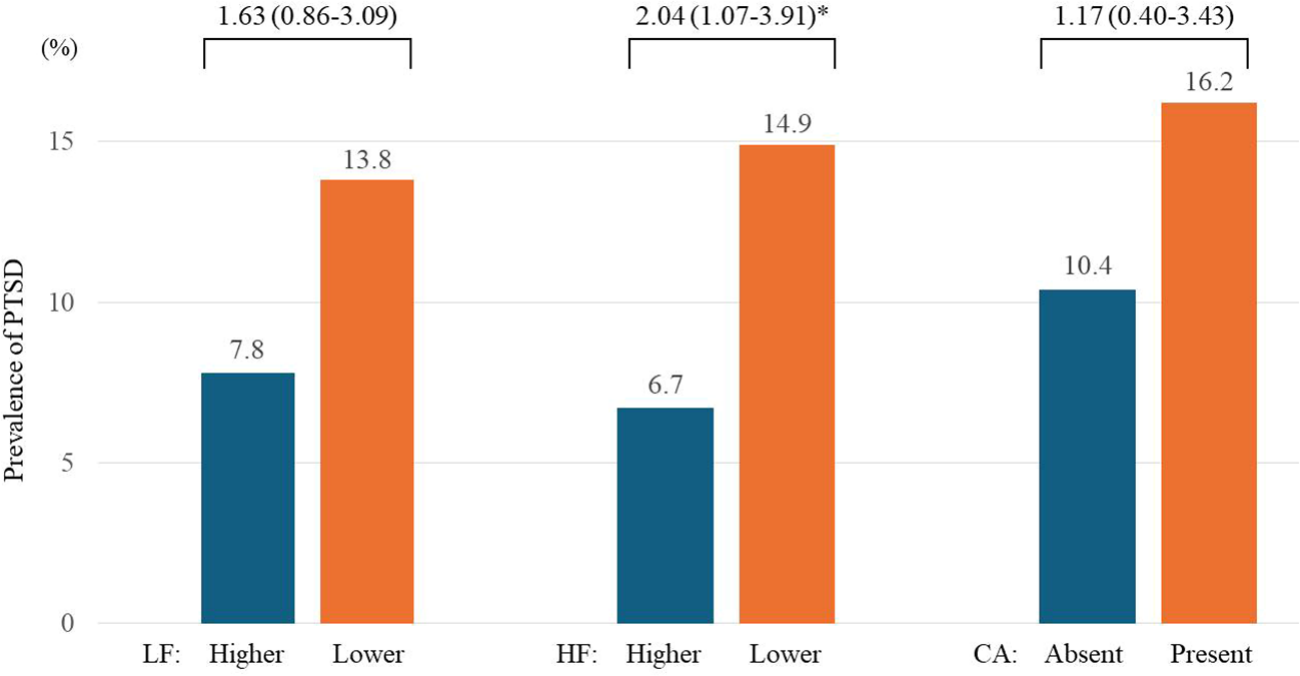
Individual associations of low frequency (LF) and high frequency (HF) bands of heart rate variability, and childhood abuse (CA) status with post-traumatic stress disorder (PTSD) over 2 years in 538 patients with physical injuries. Odds ratios (95% confidence intervals) were calculated using binary logistic regression. Associations were analyzed between higher (> 57 ms²) vs. lower (≤ 57 ms²) LF, higher (> 38 ms²) vs. lower (≤ 38 ms²) HF, and absent vs. present CA at baseline for the development of PTSD over 2 years. Adjustments were made for age, sex, previous psychiatric disorders, previous traumatic events, number of physical disorders, current smoking status, scores on the Alcohol Use Disorders Identification Test and the Hamilton Depression Rating Scale, and heart rate. ^*^P<0.05.

Childhood abuse histories were not significantly associated with either LF or HF levels (lower part of **Table 1**). The individual associations of LF, HF, and childhood abuse histories with PTSD development are illustrated in **Figure 1** and upper part of **Table 2**. Lower HF levels were significantly associated with the development of PTSD after adjusting for the identified covariates. However, LF levels and childhood abuse histories did not show significant associations with PTSD development in the adjusted model. For the logistic regression model, the Box-Tidwell procedure confirmed the assumption of linearity in the logit for all continuous variables, with all p-values greater than 0.05. Additionally, the VIF values for all independent variables were below 5, indicating that no significant multicollinearity was present. Consequently, all predictors satisfied the necessary assumptions for the logistic regression analysis.

**Table 2:** Associations of decreased low frequency (LF) and high frequency (HF) bands of heart rate variability with post-traumatic stress disorder over 2 years by childhood abuse (CA) status in 538 patients with physical injuries.

	LF			HF		
	CA	OR (95% CI)^a^	Interaction^b^ Wald	CA	OR (95% CI)^a^	Interaction^b^ Wald
Individual associations	N-A	1.21 (0.96-1.52)	N-A	N-A	1.26 (1.02-1.54)^*^	N-A
Interactive modifying	Absent	1.02 (0.74-1.37)	5.103^*^	Absent	1.01 (0.76-1.26)	5.283^*^
associations	Present	1.53 (1.26-1.98)^*^		Present	1.62 (1.32-2.07)^†^	

a Odds ratios (95% confidence intervals) were calculated using binary logistic regression for decreasing LF and HF on PTSD.

b interactive modifying associations of LF, HF, and CA on PTSD were estimated using multinomial logistic regression, adjusted for age, sex, previous psychiatric disorders, previous traumatic events, number of physical disorders, current smoking, scores on Alcohol Use Disorders Identification Test and Hamilton Depression Rating Scale, and heart rate.

^*^P<0.05; ^†^P<0.01. N-A, Not Applicable.

### Interactive modifying associations

**Figure 2:**
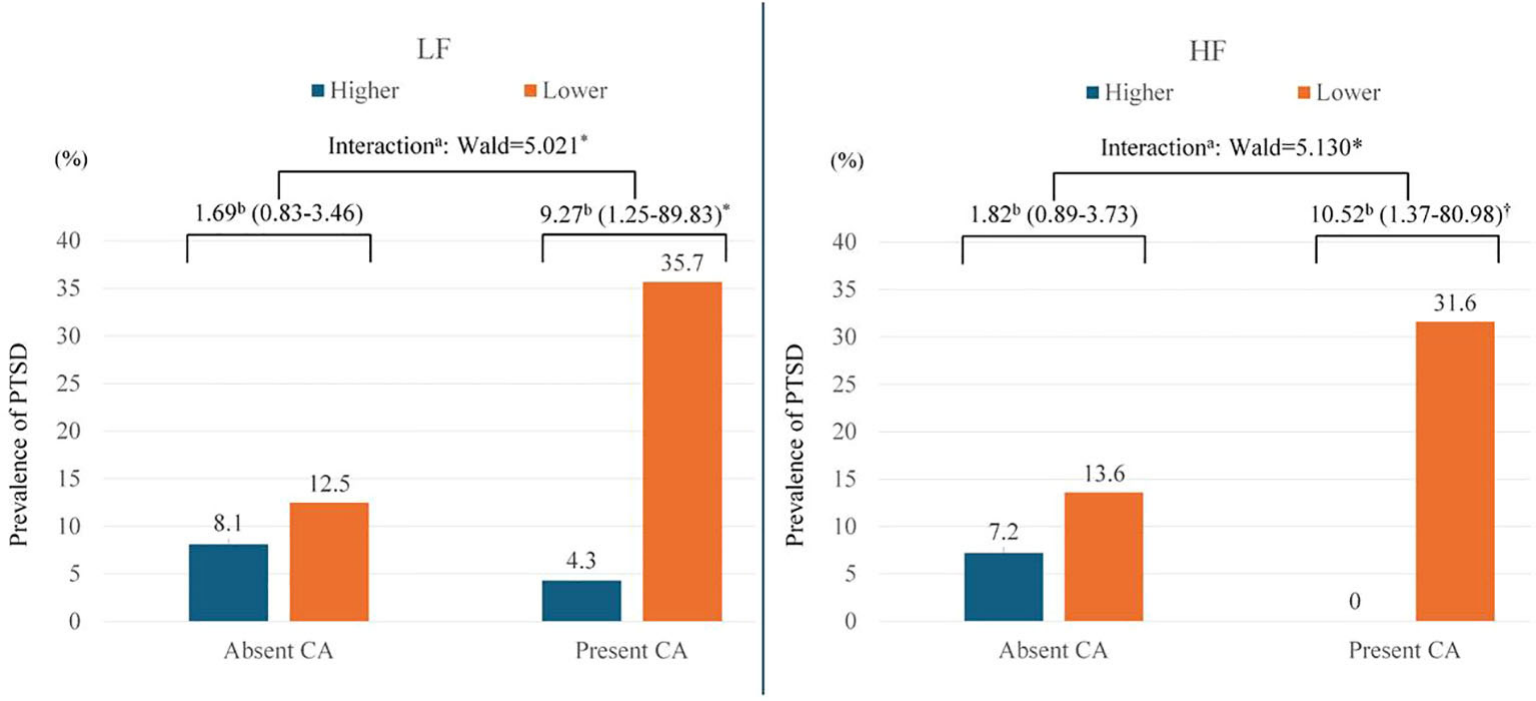
Interactive modifying associations of low frequency (LF) and high frequency (HF) bands of heart rate variability, and childhood abuse (CA) status with post-traumatic stress disorder (PTSD) over 2 years in 538 patients with physical injuries. ^a^Interactive modifying associations of LF, HF, and CA on PTSD were estimated using multinomial logistic regression; and ^b^odds ratios (95% confidence intervals) were calculated using binary logistic regression for higher (> 57 ms^2^) vs. lower (≤ 57 ms^2^) LF and for higher (> 38 ms^2^) vs. lower (≤ 38 ms^2^) HF at baseline on PTSD, adjusted for age, sex, previous psychiatric disorders, previous traumatic events, number of physical disorders, current smoking, scores on Alcohol Use Disorders Identification Test and Hamilton Depression Rating Scale, and heart rate. Both lower LF and HF levels were significantly associated with the development of PTSD in patients with CA histories, but not in those without. Significant interaction terms indicate that the effect of HRV on PTSD risk varies depending on the presence of CA histories. ^*^P<0.05; ^†^P<0.01.

The interactive modifying effects of childhood abuse histories on the relationship between LF-HF levels and PTSD development are presented in **Figure 2** and lower part of **Table 2**. Significant modifying effects were observed: both lower LF and HF levels were significantly associated with the development of PTSD in patients with childhood abuse histories, but not in those without. These results were supported by significant interaction terms, suggesting the moderating role of childhood abuse histories in the relationship between HRV components and PTSD risk. Model assumptions for the multinomial logistic regression analysis revealed similar outcomes. The Box-Tidwell procedure confirmed linearity in the logit for all continuous variables with all p-values exceeding 0.05, and the VIF values for all independent variables remained below 5, indicating an absence of significant multicollinearity. These results affirm that the multinomial model also met all necessary logistic regression assumptions.

## Discussion

The principal findings of this two-year longitudinal study reveal that lower LF and HF components of HRV significantly predict the development of PTSD. This predictive relationship is particularly pronounced in patients with a history of childhood abuse, as indicated by significant interaction terms. Interestingly, despite the lack of a direct association between childhood abuse and LF-HF HRV components, this moderating effect persists. However, this association is not observed in patients without a history of childhood abuse.

Previous research on LF-HF and PTSD has primarily utilized cross-sectional designs, showing mixed results (4–11). Direct comparisons with our prospective study are challenging, but our findings suggest HRV components are critical PTSD risk markers, especially among those with childhood abuse histories.

Several factors should be considered before drawing a conclusion. Unlike prior studies, our research did not find a direct association between childhood abuse histories and HRV outcomes or PTSD risk in isolation. This discrepancy could be attributed to various methodological differences and potential moderating variables. Firstly, previous studies often examined HRV and PTSD presence at a single point in time and categorized participants based on childhood abuse histories. These cross-sectional designs might capture the current state of HRV and PTSD without accounting for temporal dynamics and causality (12, 15). In contrast, our study evaluated HRV shortly after a significant traumatic event—physical injury—and prospectively assessed PTSD development over a two-year period. This longitudinal approach allows for a more detailed understanding of how HRV changes and PTSD symptoms evolve over time in response to trauma. Secondly, our focus on patients with moderate to severe physical injuries provides a specific context that might differ significantly from the general or varied trauma populations used in other studies. The acute stress and physiological impact of physical injuries could overshadow the effects of childhood abuse histories on HRV and PTSD risk, explaining the lack of direct associations found in our study. Moreover, we measured HRV immediately after the physical injury, providing a snapshot of the autonomic nervous system’s state in response to recent trauma. Finally, the prospective nature of our study, with follow-ups at multiple intervals, offers insights into the development of PTSD over time, something cross-sectional studies cannot provide.

Despite childhood abuse histories not being directly associated with HRV levels (**Table 1**) or PTSD development (**Figure 1**) independently in our study, and no evidence of a mediating effect being found, they significantly influenced the relationship between HRV components and PTSD risk. Several mechanisms may explain this moderating effect. First, the concept of allostatic load refers to the cumulative physiological burden of chronic stress. This concept is particularly relevant in understanding how childhood abuse leads to long-term ANS dysregulation. Individuals with a history of childhood abuse often experience prolonged activation of stress response systems, which can alter the baseline setting of autonomic functions and lead to an imbalanced physiological state (29). Over time, this chronic stress exposure can result in a hyper-reactive or hypo-reactive stress response, manifesting as altered HRV patterns, which are indicators of ANS activity. Second, the dysregulation of the ANS and the associated high allostatic load can make individuals more susceptible to subsequent stressors. This heightened sensitivity is due to the body’s compromised ability to appropriately respond to new threats or challenges. For example, a lower HRV reflects reduced parasympathetic activity and an inability to effectively manage stress responses. When individuals with such a background encounter additional traumatic events, such as physical injuries, their already strained systems are less capable of adapting, potentially triggering PTSD development (27, 28). Third, the relationship between lower HRV, higher allostatic load, and PTSD risk can be understood through the viewpoint of cardiovascular reactivity. Studies have shown that a higher allostatic load is associated with cardiovascular dysfunctions, including a reduced heart rate variability (30). This cardiovascular inflexibility may impair an individual’s ability to manage stress and recover from traumatic events, thereby increasing the risk for PTSD. Fourth, the impact of early abuse on emotional regulation and coping strategies further compounds these physiological vulnerabilities (31). Ineffective emotion regulation—often a result of early developmental trauma—can exacerbate stress responses and make individuals more reactive to stress. This increased emotional reactivity can further destabilize the ANS, making low HRV a more pronounced risk factor for PTSD following new traumatic exposures.

Conversely, patients without childhood abuse histories did not exhibit the same relationship between HRV and PTSD development. These individuals may possess more resilient autonomic and stress response systems, likely due to the absence of prolonged early-life stress exposure. This resilience could be reflected in more stable HRV patterns and an adaptive physiological response to traumatic events, which may reduce their vulnerability to PTSD compared to those with a history of childhood abuse. It is hypothesized that those without abuse histories maintain a more balanced autonomic response, enabling better physiological regulation and recovery following trauma.

There are several limitations in this study. First, this study focused exclusively on individuals with physical injuries, presenting challenges in generalizing the findings to those experiencing other types of trauma (32). Physical injuries are significant triggers for PTSD and often lead to substantial functional impairments and a diminished quality of life (33). However, caution should be exercised when extrapolating these results to populations experiencing psychological or emotional traumas, as the physiological and psychological impacts of different trauma types can vary substantially. Further research is necessary to determine if these HRV patterns are consistent across diverse trauma contexts, which would enhance the applicability of HRV monitoring in broader PTSD assessments. Second, while recruitment from a single trauma center enhanced consistency, it may limit the applicability of our findings to a broader population. Third, the completion rate for HRV evaluations was 51%, with higher Injury Severity Scores (ISS) linked to non-completion. This suggests that patients with more severe injuries were underrepresented in HRV assessments, potentially skewing the observed HRV-PTSD relationship. Fourth, while HRV was measured only at baseline, providing a critical snapshot of autonomic function shortly after physical injury, this single measurement may not fully capture the dynamic changes in HRV over time, especially as PTSD symptoms develop and evolve. The initial HRV assessment offers valuable insights into the acute stress response, which is directly relevant to the early stages of trauma response. However, PTSD is a condition that unfolds over time, and HRV may fluctuate due to a variety of factors including recovery processes, treatment interventions, and the natural progression of PTSD. Therefore, future research should consider incorporating repeated HRV measurements to track these changes more comprehensively. Fifth, the relatively small sample size for individuals with childhood abuse histories may limit the statistical power of our findings and impact the robustness and generalizability of the interaction effects observed. This constraint raises concerns about the strength of the conclusions that can be drawn regarding the moderating role of childhood abuse on the HRV-PTSD relationship. The limited sample size may result in lower confidence in detecting true effects and could potentially bias the estimates, making it challenging to apply these results broadly across different populations. Future studies are encouraged to replicate these findings with larger, more diverse samples to validate and extend our understanding of these complex interactions. Lastly, while telephone interviews for follow-up assessments have been validated and are practical for wide-reaching studies (34), their reliance may still compromise the depth and precision achievable through in-person evaluations. This method, though necessary under certain circumstances, could potentially influence the reliability and subtlety of the data collected, especially in capturing complex changes in PTSD symptoms over time. Therefore, future studies might consider incorporating a combination of in-person and remote assessment techniques to enhance data accuracy and reliability.

The study’s strengths include its two-year longitudinal design and a substantial cohort, which allow for a comprehensive analysis over time. By recruiting consecutively from the entire population of recently injured patients, the study minimizes selection bias and ensures a more representative sample. Regular follow-up evaluations help reduce inconsistencies related to the timing of assessments. The rigorous research protocol applied throughout the study ensures uniform data collection and evaluation procedures, enhancing the reliability and validity of the findings. Furthermore, the extensive collection of potential baseline covariates enables a thorough analysis of various influencing factors. The high follow-up rates and lack of significant selective attrition support the robustness and credibility of the study’s results.

In conclusion, our study provides important insights into the role of HRV and childhood abuse histories in predicting PTSD development. Lower LF and HF HRV components are significant predictors of PTSD only in patients with childhood abuse histories, suggesting the need to consider childhood abuse histories in PTSD risk assessments. For public health, identifying individuals with a history of childhood abuse and monitoring their HRV allows health professionals to better target resources and interventions to those at higher risk for PTSD. This proactive approach can help mitigate the long-term psychological and physiological effects of trauma, ultimately reducing the burden of PTSD on healthcare systems. In the clinical context, it is crucial that healthcare providers screen for childhood abuse histories in physically injured patients and consider incorporating HRV monitoring into the assessment process. Specifically, interventions designed to improve HRV, such as biofeedback, mindfulness practices, and physical exercise, could be particularly beneficial for patients with a history of childhood abuse. These interventions are known to enhance autonomic regulation, potentially reducing PTSD risk by stabilizing HRV (35). Furthermore, future research should not only continue to dissect the mechanisms linking childhood abuse, HRV, and PTSD but also rigorously evaluate the efficacy of HRV-based interventions. Longitudinal studies that track changes in HRV over time and correlate these changes with PTSD outcomes will be particularly insightful. Such studies could provide crucial evidence on how early interventions targeting HRV improvements might alter PTSD trajectories, especially in vulnerable populations. This approach will help clarify these complex relationships and identify additional moderating factors, thereby enhancing personalized treatment strategies for PTSD.

## Data Availability

The original contributions presented in the study are included in the article/**Supplementary Material**. Further inquiries can be directed to the corresponding author.
