# Supplementary material for: Impacts of heart rate variability on post-traumatic stress disorder risks after physical injuries: amplification with childhood abuse histories
Source: Front Psychiatry. 2024 Dec 19;15:1474650. doi: 10.3389/fpsyt.2024.1474650 (PMC11694026; doi:10.3389/fpsyt.2024.1474650)
Supplement: Supplementary file 1 [file DataSheet1.docx]

Online Supplementary Materials

**Figure S1.** Patient flow and prevalence of post-traumatic stress disorder (PTSD).

**Table S1.** Patient data at baseline by completion of the heart rate variability (HRV) evaluation in 1,142 patients with physical injuries.

**Table S2.** Baseline characteristics by post-traumatic stress disorder (PTSD) diagnosis over 2 years in 538 patients with physical injuries.

**Table S3.** Baseline characteristics by lower (≤ 38 ms^2^) vs. higher (> 38 ms^2^) high frequency (HF) component of heart rate variability in 538 patients with physical injuries.

**Figure S1.** Patient flow and prevalence of post-traumatic stress disorder (PTSD).


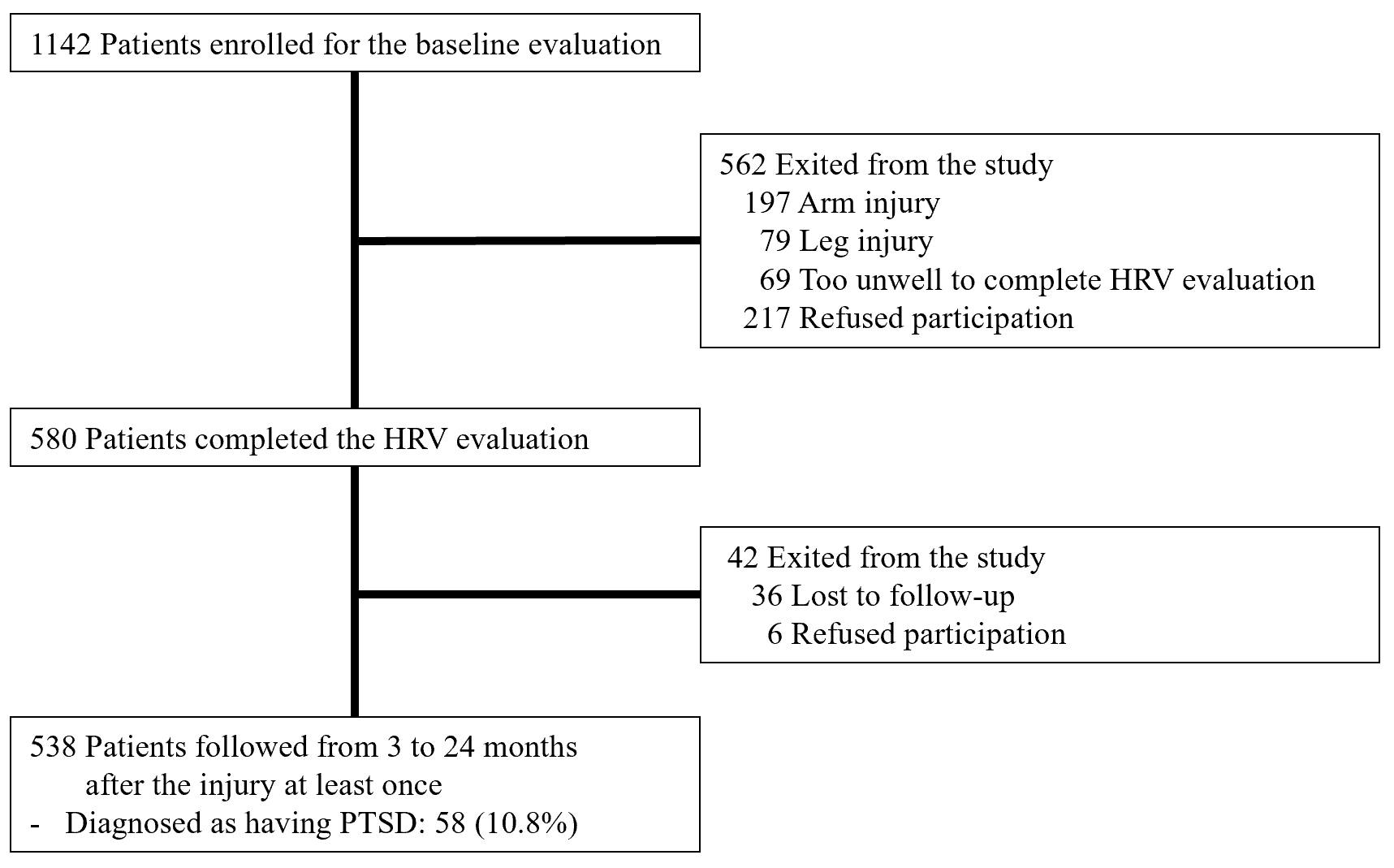


| **Table S1.** Patient data at baseline by completion of the heart rate variability (HRV) evaluation in 1,142 patients with physical injuries. | | | | |
| --- | --- | --- | --- | --- |
|  | Completed HRV  (N=580) | Uncompleted HRV  (N=562) | Statistical coefficients | P-value^a^ |
| **Socio-demographic characteristics** |  |  |  |  |
| Age, mean (SD) years | 57.2 (16.8) | 56.4 (17.6) | t=+0.750 | 0.454 |
| Sex, N (%) female | 182 (31.4) | 177 (31.5) | χ^2^=0.002 | 0.967 |
| Education, mean (SD) years | 10.6 (4.3) | 10.8 (3.9) | t=-0.516 | 0.606 |
| Marital status, N (%) unmarried | 191 (32.9) | 191 (34.0) | χ^2^=0.143 | 0.706 |
| Living alone, N (%) | 89 (15.3) | 88 (15.7) | χ^2^=0.021 | 0.884 |
| Unemployed status, N (%) | 100 (17.2) | 120 (21.4) | χ^2^=3.101 | 0.078 |
| **Pre-trauma characteristics** |  |  |  |  |
| Previous psychiatric disorders, N (%) | 49 (8.4) | 37 (6.6) | χ^2^=1.425 | 0.233 |
| Previous traumatic events, N (%) | 29 (5.0) | 18 (3.2) | χ^2^=2.336 | 0.126 |
| Any childhood abuse, N (%) | 40 (6.9) | 30 (5.3) | χ^2^=1.205 | 0.272 |
| Physical disorders, mean (SD) numbers | 2.0 (2.1) | 1.9 (2.0) | t=+1.063 | 0.288 |
| Current smoker, N (%) | 156 (26.9) | 162 (28.8) | χ^2^=0.529 | 0.467 |
| AUDIT, mean (SD) scores | 10.3 (10.1) | 10.0 (10.0) | t=+0.382 | 0.702 |
| Body mass index, mean (SD) | 23.6 (3.4) | 23.5 (3.4) | t=+0.144 | 0.886 |
| **Trauma related characteristics** |  |  |  |  |
| Injury type, N (%) intentional | 53 (9.1) | 45 (8.0) | χ^2^=0.465 | 0.495 |
| Injury Severity Score, mean (SD) scores | 14.1 (5.3) | 15.0 (6.3) | t=-2.747 | **0.006** |
| Glasgow Coma Scale, mean (SD) scores | 14.9 (0.6) | 14.8 (0.8) | t=+1.415 | 0.157 |
| Got surgery for the injury, N (%) | 291 (50.2) | 312 (55.5) | χ^2^=2.270 | 0.071 |
| **Peri-trauma assessment scales and measurements**, mean (SD) |  |  |  |  |
| CAPS-5 | 11.0 (11.5) | 11.3 (12.6) | t=-0.451 | 0.652 |
| HAM-A | 4.7 (4.6) | 4.7 (5.1) | t=-0.070 | 0.944 |
| HAM-D | 6.3 (5.4) | 6.2 (5.8) | t=+0.327 | 0.744 |
| Systolic blood pressure, mmHg | 119.1 (14.1) | 120.0 (13.3) | t=-1.070 | 0.285 |
| Diastolic blood pressure, mmHg | 72.0 (9.2) | 72.5 (9.1) | t=-0.920 | 0.358 |
| Heart rate per minute | 78.7 (11.1) | 78.0 (11.0) | t=+1.129 | 0.259 |
| ^a^ t-tests or χ^2^ tests, as appropriate between patients with and without 24-months follow-up evaluation.  AUDIT: Alcohol Use Disorders Identification Test; CAPS-5: Clinician Administered PTSD Scale for Diagnostic and Statistical Manual of mental disorders, 5^th^ edition; HAMA: Hamilton Anxiety Rating Scale; HAMD: Hamilton Depression Rating Scale. | | | | |

| **Table S2.** Baseline characteristics by post-traumatic stress disorder (PTSD) diagnosis over 2 years in 538 patients with physical injuries. | | | | |
| --- | --- | --- | --- | --- |
|  | Present  PTSD (N=58) | Absent  PTSD  (N=480) | Statistical coefficients | P-value^a^ |
| **Socio-demographic characteristics** |  |  |  |  |
| Age, mean (SD) years | 54.1 (15.8) | 57.4 (17.1) | t=-1.408 | 0.160 |
| Sex, N (%) female | 25 (43.1) | 144 (30.0) | χ^2^=4.124 | **0.042** |
| Education, mean (SD) years | 11.9 (3.5) | 10.5 (4.3) | t=+2.819 | **0.006** |
| Marital status, N (%) unmarried | 17 (29.3) | 161 (33.5) | χ^2^=0.418 | 0.518 |
| Living alone, N (%) | 5 (8.6) | 74 (15.4) | χ^2^=1.908 | 0.167 |
| Unemployed status, N (%) | 9 (15.5) | 79 (16.5) | χ^2^=0.033 | 0.855 |
| **Pre-trauma characteristics** |  |  |  |  |
| Previous psychiatric disorders, N (%) | 11 (19.0) | 30 (6.3) | χ^2^=11.884 | **0.001** |
| Previous traumatic events, N (%) | 8 (13.8) | 21 (4.4) | χ^2^=9.000 | **0.003** |
| Physical disorders, mean (SD) numbers | 1.6 (1.9) | 2.1 (2.1) | t=-1.178 | 0.239 |
| Current smoker, N (%) | 15 (25.9) | 125 (26.0) | χ^2^=0.001 | 0.977 |
| AUDIT, mean (SD) scores | 8.7 (10.0) | 10.4 (10.0) | t=-1.207 | 0.228 |
| Body mass index, mean (SD) | 23.8 (3.7) | 23.6 (3.4) | t=+0.341 | 0.733 |
| **Trauma related characteristics** |  |  |  |  |
| Injury type, N (%) intentional | 4 (6.9) | 47 (9.8) | χ^2^=0.505 | 0.477 |
| Injury Severity Score, mean (SD) scores | 13.9 (4.9) | 14.0 (5.3) | t=-0.217 | 0.829 |
| Glasgow Coma Scale, mean (SD) scores | 14.8 (0.7) | 14.9 (0.6) | t=-0.628 | 0.530 |
| Got surgery for the injury, N (%) | 32 (55.2) | 236 (49.2) | χ^2^=0.747 | 0.388 |
| **Peri-trauma assessment scales and measurements**, mean (SD) |  |  |  |  |
| HAMA | 9.5 (6.0) | 4.1 (4.1) | t=+6.697 | **<0.001** |
| HAMD | 11.4 (6.4) | 5.7 (5.0) | t=+6.557 | **<0.001** |
| Systolic blood pressure, mmHg | 117.8 (11.9) | 119.3 (14.4) | t=-0.780 | 0.435 |
| Diastolic blood pressure, mmHg | 70.7 (9.9) | 72.1 (9.1) | t=-1.123 | 0.262 |
| Heart rate per minute | 80.3 (10.6) | 78.5 (11.1) | t=+1.210 | 0.227 |
| ^a^ t-tests or χ^2^ tests, as appropriate between patients with and without 24-months follow-up evaluation.  AUDIT: Alcohol Use Disorders Identification Test; HAMA: Hamilton Anxiety Rating Scale; HAMD: Hamilton Depression Rating Scale. | | | | |

| **Table S3.** Baseline characteristics by lower (≤ 38 ms^2^) vs. higher (> 38 ms^2^) high frequency (HF) component of heart rate variability in 538 patients with physical injuries. | | | | |
| --- | --- | --- | --- | --- |
|  | Lower HF (N=269) | Higher HF  (N=269) | Statistical coefficients | P-value^a^ |
| **Socio-demographic characteristics** |  |  |  |  |
| Age, mean (SD) years | 58.6 (15.8) | 55.4 (17.9) | t=+2.183 | **0.019** |
| Sex, N (%) female | 87 (32.3) | 82 (30.5) | χ^2^=0.216 | 0.642 |
| Education, mean (SD) years | 10.3 (4.2) | 11.0 (4.2) | t=-1.866 | 0.063 |
| Marital status, N (%) unmarried | 82 (30.5) | 96 (35.7) | χ^2^=1.646 | 0.200 |
| Living alone, N (%) | 34 (12.6) | 45 (16.7) | χ^2^=1.795 | 0.180 |
| Unemployed status, N (%) | 36 (13.4) | 52 (19.3) | χ^2^=3.478 | 0.062 |
| **Pre-trauma characteristics** |  |  |  |  |
| Previous psychiatric disorders, N (%) | 21 (7.8) | 20 (7.4) | χ^2^=0.026 | 0.871 |
| Previous traumatic events, N (%) | 17 (6.3) | 12 (4.5) | χ^2^=0.911 | 0.340 |
| Physical disorders, mean (SD) numbers | 2.1 (2.1) | 1.9 (2.1) | t=+0.972 | 0.332 |
| Current smoker, N (%) | 64 (23.8) | 76 (28.3) | χ^2^=1.390 | 0.238 |
| AUDIT, mean (SD) scores | 10.2 (10.0) | 10.3 (10.0) | t=-0.112 | 0.911 |
| Body mass index, mean (SD) | 23.6 (3.3) | 23.6 (3.7) | t=+0.088 | 0.930 |
| **Trauma related characteristics** |  |  |  |  |
| Injury type, N (%) intentional | 29 (10.8) | 22 (8.2) | χ^2^=1.061 | 0.303 |
| Injury Severity Score, mean (SD) scores | 14.1 (5.3) | 13.9 (5.2) | t=+0.533 | 0.594 |
| Glasgow Coma Scale, mean (SD) scores | 14.9 (0.6) | 14.9 (0.6) | t=-0.538 | 0.560 |
| Got surgery for the injury, N (%) | 132 (49.1) | 136 (50.6) | χ^2^=0.119 | 0.730 |
| **Peri-trauma assessment scales and measurements**, mean (SD) |  |  |  |  |
| HAMA | 5.2 (5.1) | 4.2 (4.2) | t=+2.715 | **0.007** |
| HAMD | 6.9 (5.7) | 5.7 (5.0) | t=+2.732 | **0.007** |
| Systolic blood pressure, mmHg | 119.5 (13.6) | 118.8 (14.6) | t=+0.426 | 0.592 |
| Diastolic blood pressure, mmHg | 72.0 (9.3) | 71.9 (9.1) | t=+0.122 | 0.903 |
| Heart rate per minute | 81.5 (10.7) | 75.8 (10.7) | t=+6.227 | **<0.001** |
| ^a^ t-tests or χ^2^ tests, as appropriate between patients with and without 24-months follow-up evaluation.  AUDIT: Alcohol Use Disorders Identification Test; HAMA: Hamilton Anxiety Rating Scale; HAMD: Hamilton Depression Rating Scale. | | | | |
